# Baseline predictors of in-hospital mortality after acute traumatic spinal cord injury: data from a level I trauma center

**DOI:** 10.1038/s41598-022-15469-z

**Published:** 2022-07-06

**Authors:** Christian Blex, Martin Kreutzträger, Johanna Ludwig, Claus Peter Nowak, Jan M. Schwab, Tom Lübstorf, Axel Ekkernkamp, Marcel A. Kopp, Thomas Liebscher

**Affiliations:** 1 Clinical and Experimental Spinal Cord Injury Research (Neuroparaplegiology), Department of Neurology With Experimental Neurology, Charité – Universitätsmedizin Berlin, corporate member of Freie Universität Berlin, Humboldt-Universität zu Berlin, and Berlin Institute of Health, Charitéplatz 1, 10117 Berlin, Germany; 2 QUEST-Center for Transforming Biomedical Research, Berlin Institute of Health (BIH), Anna-Louisa-Karsch-Str. 2, 10178 Berlin, Germany; 3 Treatment Center for Spinal Cord Injuries, Trauma Hospital Berlin, Warener Str. 7, 12683 Berlin, Germany; 4 Clinic for Trauma Surgery and Orthopedics, Trauma Hospital Berlin, Warener Str. 7, 12683 Berlin, Germany; 5 Institute of Biometry and Clinical Epidemiology, Charité – Universitätsmedizin Berlin, Corporate Member of Freie Universität Berlin, Humboldt-Universität zu Berlin, and Berlin Institute of Health, Charitéplatz 1, 10117 Berlin, Germany; 6 Berlin Institute of Health (BIH), Anna-Louisa-Karsch-Str. 2, 10178 Berlin, Germany; 7 Spinal Cord Injury Medicine (Neuroparaplegiology), Department of Neurology, The Ohio State University, Wexner Medical Center, Columbus, OH USA; 8 Belford Center for Spinal Cord Injury, Departments of Neuroscience and Physical Medicine and Rehabilitation, The Neurological Institute, The Ohio State University, Wexner Medical Center, Columbus, OH USA; 9 Department of Traumatology, University of Greifswald, Sauerbruchstraße, 17491 Greifswald, Germany

**Keywords:** Spinal cord diseases, Trauma, Outcomes research, Epidemiology

## Abstract

Comorbidity scores are important predictors of in-hospital mortality after traumatic spinal cord injury (tSCI), but the impact of specific pre-existing diseases is unknown. This retrospective cohort study aims at identifying relevant comorbidities and explores the influence of end-of-life decisions. In-hospital mortality of all patients admitted to the study center after acute tSCI from 2011 to 2017 was assessed. A conditional inference tree analysis including baseline data, injury characteristics, and Charlson Comorbidity Index items was used to identify crucial predictors. End-of-life decisions were recorded. Three-hundred-twenty-one patients were consecutively enrolled. The median length of stay was 95.7 days (IQR 56.8–156.0). During inpatient care, 20 patients (6.2%) died. These patients were older (median: 79.0 (IQR 74.7–83.2) vs. 55.5 (IQR 41.4–72.3) years) and had a higher Charlson Comorbidity Index score (median: 4.0 (IQR 1.75–5.50) vs. 0.0 (IQR 0.00–1.00)) compared to survivors. Pre-existing kidney or liver disease were identified as relevant predictors of in-hospital mortality. End-of-life decisions were observed in 14 (70.0%) cases. The identified impairment of kidney and liver, important for drug metabolism and elimination, points to the need of careful decisions on pharmaceutical treatment regimens after tSCI. Appropriate reporting of end-of-life decisions is required for upcoming studies.

## Introduction

The life expectancy of people suffering from traumatic spinal cord injury (tSCI) has been increasing in high-income countries over the last decades^1^. Improvements in prevention, pre-hospital- and inpatient care and rehabilitation added to this beneficial effect. Nevertheless, affected patients are still more likely to die prematurely compared to people without tSCI^2–5^. Furthermore, the mortality during the first year after injury has a strong impact on the overall survival rate after tSCI^6–8^ spanning clinical acute and rehabilitation care of tSCI patients. The changing epidemiology of tSCI is characterized by an increasing number of geriatric patients and especially elderly tSCI-patients suffer from a higher rate of complications and show an increased mortality rate during the first year post-injury^9–11^, reaching mortality rates as high as 38.6%^12^.

Besides age, other demographic-, injury-, and treatment characteristics^7,13^, as well as pre-existing comorbidities^11,14–17^ have been identified as clinical predictors of mortality after tSCI. However, up to now there have been no analyses on specific pre-existing comorbidities and their influence on in-hospital mortality after tSCI. Filling this gap of knowledge may lead to improvements in medical treatment and health-care strategies for relevant patient groups. Additionally, end-of-life decisions contribute to mortality after tSCI^18^, but have rarely been considered previously within the limited amount of available literature.

Within this exploratory study, observational tSCI-study data from a German level I trauma center is analyzed in order to identify risk factors of in-hospital mortality. Special emphasis is put on pre-admission comorbidities and injury characteristics that may allow an early identification of patients at risk, supported by information on clinical complications and cause of death including end-of-life decisions. Additionally, the results will supplement the scarce information for in-hospital mortality after tSCI.

## Results

From January 2011 to December 2017, 321 patients were treated for acute tSCI at the Trauma Hospital Berlin. The median length of in-hospital stay was 95.7 days and 309 of 321 patients (96.3%) received a spinal surgery. Twenty of the 321 patients (6.2%) died during in hospital treatment. In-hospital deceased patients were older, had a higher Charlson Comorbidity Index (CCI), more frequently suffered fall related injuries and were more often secondary referrals from other hospitals compared to survivors (Table 1). Although the length of in-hospital stay was shorter for deceased patients, the frequency and length of intensive-care-unit (ICU)-stay were increased compared to survivors. While we see no considerable differences of American Spinal Injury Association Impairment Scale (AIS), neurological level of injury (NLI), body mass index (BMI), gender and time to admission in terms of the summary measures (percentage, medians, IQR) presented in Table 1, the frequency of complete tSCI injuries and the percentage of female patients was higher in the deceased patient group.

**Table 1:** Patient characteristics.

Variable	All patients n = 321	Survivors n = 301	Deceased n = 20	p-value
Age (median [IQR])	57.3 [42.6; 73.9]	55.5 [41.4; 72.3]	79.0 [74.7; 83.2]	< 0.001
**Gender**				0.291
Male	241 (75.1%)	228 (75.7%)	13 (65.0%)	
Female	80 (24.9%)	73 (24.3%)	7 (35.0%)	
**Etiology**				0.052
Fall	168 (52.3%)	150 (49.8%)	18 (90.0%)	
Traffic	90 (28.0%)	88 (29.2%)	2 (10.0%)	
Sports & recreation	29 (9.0%)	29 (9.6%)	0 (0.0%)	
Falling object	11 (3.4%)	11 (3.7%)	0 (0.0%)	
Suicide attempt	20 (6.2%)	20 (6.6%)	0 (0.0%)	
Violence	3 (0.9%)	3 (1.0%)	0 (0.0%)	
BMI (median [IQR])*	24.9 [23.1; 27.8]	24.9 [23.1; 27.8]	25.4 [23.5; 27.0]	0.992
AIS at admission*				0.138
A	137 (43.6%)	126 (42.7%)	11 (57.9%)	
B	25 (8.0%)	24 (8.1%)	1 (5.3%)	
C	38 (12.1%)	34 (11.5%)	4 (21.1%)	
D	114 (36.3%)	111 (37.6%)	3 (15.8%)	
NLI at admission*				0.771
Cervical	174 (55.6%)	162 (55.1%)	12 (63.2%)	
Thoracic	71 (22.7%)	68 (23.1%)	3 (15.8%)	
Lumbosacral	68 (21.7%)	64 (21.8%)	4 (21.1%)	
Charlson Comorbidity Index (median [IQR])	0.00 [0.00; 2.00]	0.00 [0.00; 1.00]	4.00 [1.75; 5.50]	< 0.001
**Spinal surgery**				0.208
No surgery	12 (3.7%)	10 (3.3%)	2 (10.0%)	
1 surgery	126 (39.3%)	116 (38.5%)	10 (50.0%)	
2 surgeries	145 (45.2%)	139 (46.2%)	6 (30.0%)	
3 surgeries	38 (11.8%)	36 (12.0%)	2 (10.0%)	
**Admission**				0.016
Primary referral	139 (43.3%)	136 (45.2%)	3 (15.0%)	
Secondary referral	182 (56.7%)	165 (54.8%)	17 (85.0%)	
Time to admission: (median [IQR], hours)	5.07 [1.33; 120]	4.97 [1.33; 120]	6.30 [2.73; 270]	0.455
Length of stay: (median [IQR], days)	95.7 [56.8; 156]	96.6 [58.3; 156]	85.2 [13.6; 105]	0.039
ICU stay	247 (76.9%)	228 (75.7%)	19 (95.0%)	0.054
Length of ICU stay: (median [IQR], days)	6.00 [1.00; 26.0]	6.00 [0.81; 25.8]	19.0 [3.88; 43.5]	0.014

Data are provided for all tSCI-patients and subgroups of survivors and deceased patients. Categorial variables are displayed as absolute (N) and relative frequencies (%). *AIS* ASIA impairment scale, *BMI* body mass index, *ICU* intensive care unit, *IQR* interquartile range, *NLI* neurological level of injury; *Determination at admission impossible in n = 7 (AIS) and n = 8 (NLI) cases; BMI data of 10 survivors are missing.

### Age and Charlson Comorbidity Index

**Figure 1:**
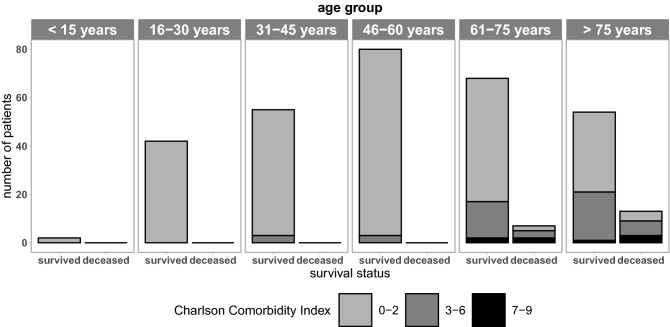
Distribution of age and comorbidities. Number of survivors and in-hospital deceased patients with acute traumatic spinal cord injury and distribution of age and Charlson Comorbidity Index (CCI) within the study population.

The age distribution of the n = 321 patients reported in 15 year age groups^19^ showed a peak of tSCI at the ‘46–60 years’ age group and the number of patients in the ‘61–75 years’ and ‘> 75 years’ groups remained high (Fig. 1). The preexisting comorbidities of the patients, assessed by the CCI, were in the range of 0–2 points for 263 patients. The number of patients obtaining a CCI of 3–6 was low within the ‘31–45 years’ and ‘46–60 years’ group and increased considerably in patients aged 61–75 or 75 years and older. In addition, patients with a CCI of 7–9 were solely observed in the two highest age groups.

The observed in-hospital mortality was restricted to patients aged 61 years or older and the major fraction of the deceased patients obtained a CCI of three or higher. Additionally, subjects with a CCI of 7–9 were more frequent in the deceased patient population.

### Comorbidities and concomitant injuries

Explorative comparison of single items of the CCI between both groups revealed higher relative frequencies within the deceased patient group, applying to all items, except for ‘myocardial infarction’ and ‘any tumor’ (Table 2). The items ‘connective tissue disease’, ‘leukemia’, ‘lymphoma’ and ‘AIDS’ were not present in the in-hospital deceased patient group. Differences of CCI-items revealing higher relative frequencies in in-hospital deceased patients were observed for the items ‘congestive heart failure’, ‘kidney disease’, ‘liver disease’, ‘metastatic tumor’, ‘cerebrovascular disease’, ‘chronic pulmonary disease’ and ‘diabetes mellitus’.

**Table 2:** Pre-existing comorbidities based on CCI-items.

Pre-existing disease	All patients n = 321	Survivors n = 301	Deceased n = 20	p-value
Myocardial infarction	19 (5.9%)	18 (6.0%)	1 (5.0%)	1.000
Congestive heart failure	54 (16.8%)	42 (14.0%)	12 (60.0%)	< 0.001
Peripheral vascular disease	13 (4.1%)	11 (3.7%)	2 (10.0%)	0.190
Cerebrovascular disease	14 (4.4%)	10 (3.3%)	4 (20.0%)	0.007
Dementia	16 (5.0%)	13 (4.3%)	3 (15.0%)	0.069
Chronic pulmonary disease	26 (8.1%)	21 (7.0%)	5 (25.0%)	0.016
Connective tissue disease	3 (0.9%)	3 (1.0%)	0 (0.0%)	1.000
Peptic ulcer	7 (2.2%)	5 (1.7%)	2 (10.0%)	0.064
Liver disease	10 (3.1%)	6 (2.0%)	4 (20.0%)	0.002
Diabetes mellitus	46 (14.3%)	40 (13.3%)	6 (30.0%)	0.050
Hemiplegia	4 (1.3%)	3 (1.0%)	1 (5.0%)	0.228
Kidney disease (moderate/severe)	24 (7.5%)	16 (5.3%)	8 (40.0%)	< 0.001
Any tumor	25 (7.8%)	24 (8.0%)	1 (5.0%)	1.000
Lymphoma	2 (0.6%)	2 (0.7%)	0 (0.0%)	1.000
Metastatic tumor	5 (1.6%)	2 (0.7%)	3 (15.0%)	0.002
AIDS	2 (0.6%)	2 (0.7%)	0 (0.0%)	1.000

Absolute (N) and relative frequencies (%) of pre-existing comorbidities are provided. Divergent from Charlson Comorbidity Index items applicable for its calculation^19^, ‘liver disease’ includes mild and moderate-severe cases and ‘diabetes mellitus’ combines cases with and without end-organ damage. There were no cases of leukemia. *AIDS* acquired immune deficiency syndrome.

Besides pre-existing comorbidities, concomitant injuries may contribute to in-hospital mortality. Therefore, the pattern of concomitant injuries was analyzed (Table 3). Traumatic brain injury (TBI), injuries to chest or abdomen, fractures of the sternal ribs or the upper extremities and injuries of the vertebral arteria were observed in the in-hospital deceased patient group. Compared to survivors, there were no obvious differences in the relative frequency of concomitant injuries. Moreover, all but injuries to the vertebral arteria showed lower relative frequencies in the deceased patient group.

**Table 3:** Concomitant injuries.

Concomitant injuries	All patients n = 321	Survivors n = 301	Deceased n = 20	p-value
Skull fracture	24 (7.5%)	24 (8.0%)	0 (0.0%)	0.381
Sternal rib fracture	102 (31.8%)	97 (32.2%)	5 (25.0%)	0.671
Upper extremity fracture	48 (15.0%)	47 (15.6%)	1 (5.0%)	0.330
Lower extremity fracture	43 (13.4%)	43 (14.3%)	0 (0.0%)	0.088
Traumatic brain injury	73 (22.7%)	70 (23.3%)	3 (15.0%)	0.582
Vertebral arteria injury*	32 (10.0%)	29 (9.7%)	3 (15.0%)	0.436
Chest injury	91 (28.3%)	89 (29.6%)	2 (10.0%)	0.104
Abdominal trauma	27 (8.4%)	26 (8.6%)	1 (5.0%)	1.000
Large vessel injury	11 (3.4%)	11 (3.7%)	0 (0.0%)	1.000

Absolute (N) and relative frequencies (%) of concomitant injuries. *There is one missing value for vertebral arteria injury in the survivor group, because radiologic analysis was impossible.

### Baseline predictors of in-hospital mortality

**Figure 2:**
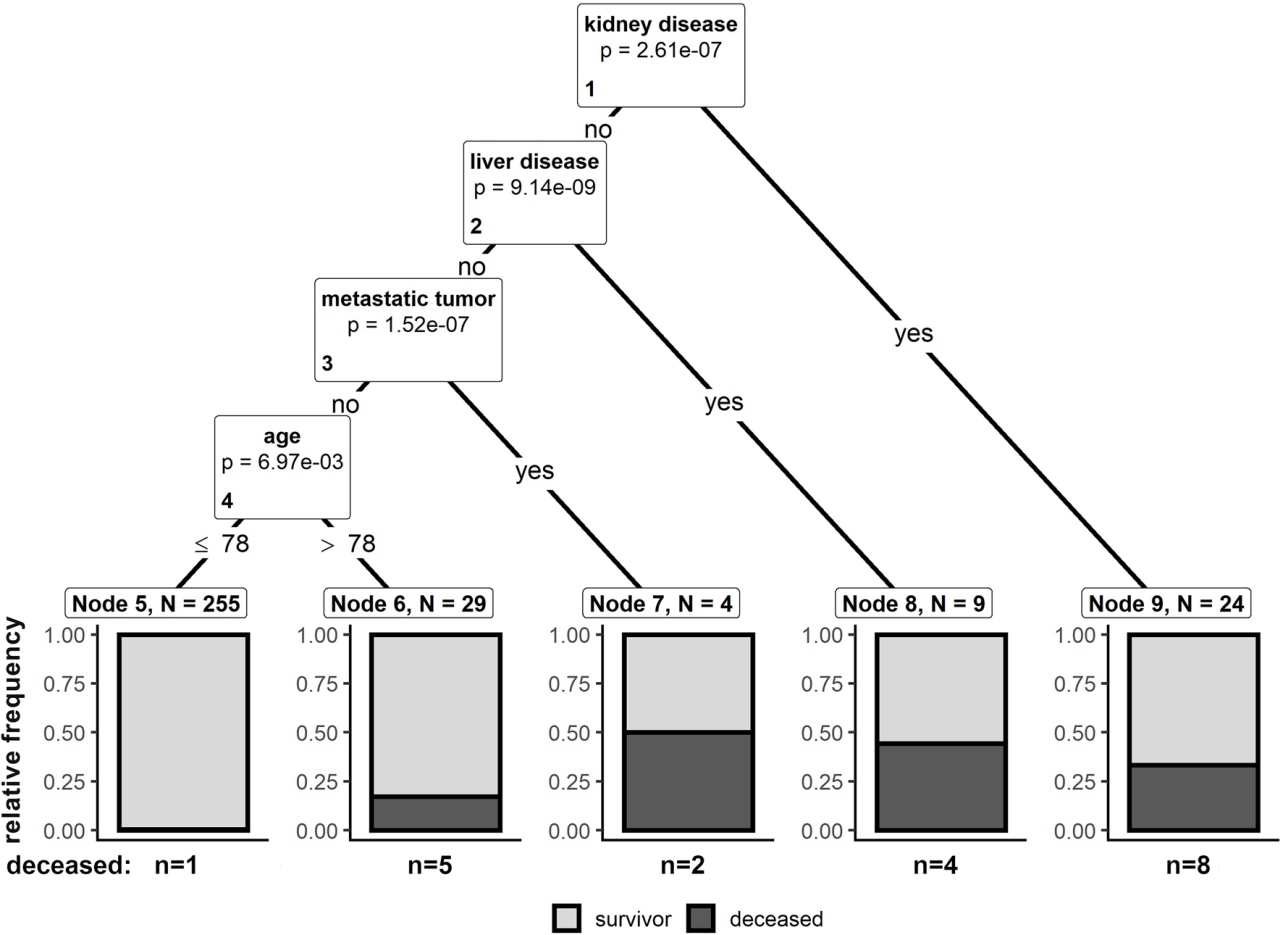
Conditional inference tree. The tree was fitted based on age, gender, pre-injury comorbidities and injury characteristics to identify predictors of in-hospital mortality. For node 1–4, the Bonferroni-adjusted p*-*values are given. The terminal nodes 5–9 provide information on the number of individuals (N) of the node and the relative frequency of deceased patients per node (grey bar). Absolute frequencies of deceased patients (n) per node are provided below the bar plot.

In order to identify patients at risk at the earliest, clinical information, individual pre-existing comorbidities and injury characteristics available at hospital admission were used as explanatory variables in a conditional inference tree approach. The tree was fitted using age as continuous variable and gender, ASIA impairment scale, NLI, relevant CCI-items and concomitant injuries as binary variables. Figure 2 shows the resulting conditional inference tree. Three pre-existing diseases were identified as the most relevant predictors associated with in-hospital mortality after tSCI, specifically kidney disease, liver disease and metastatic tumor. Furthermore, age was selected as a fourth predictor, splitting the remaining patient population into two groups at an age of 78 years.

In addition, multiple logistic regression analyses were carried out providing odds-ratios as quantitative measures for the simultaneous effects of the respective predictors on in-hospital mortality, although these have to be taken with considerable caution due to the low number of in-hospital deaths.

**Figure 3:**
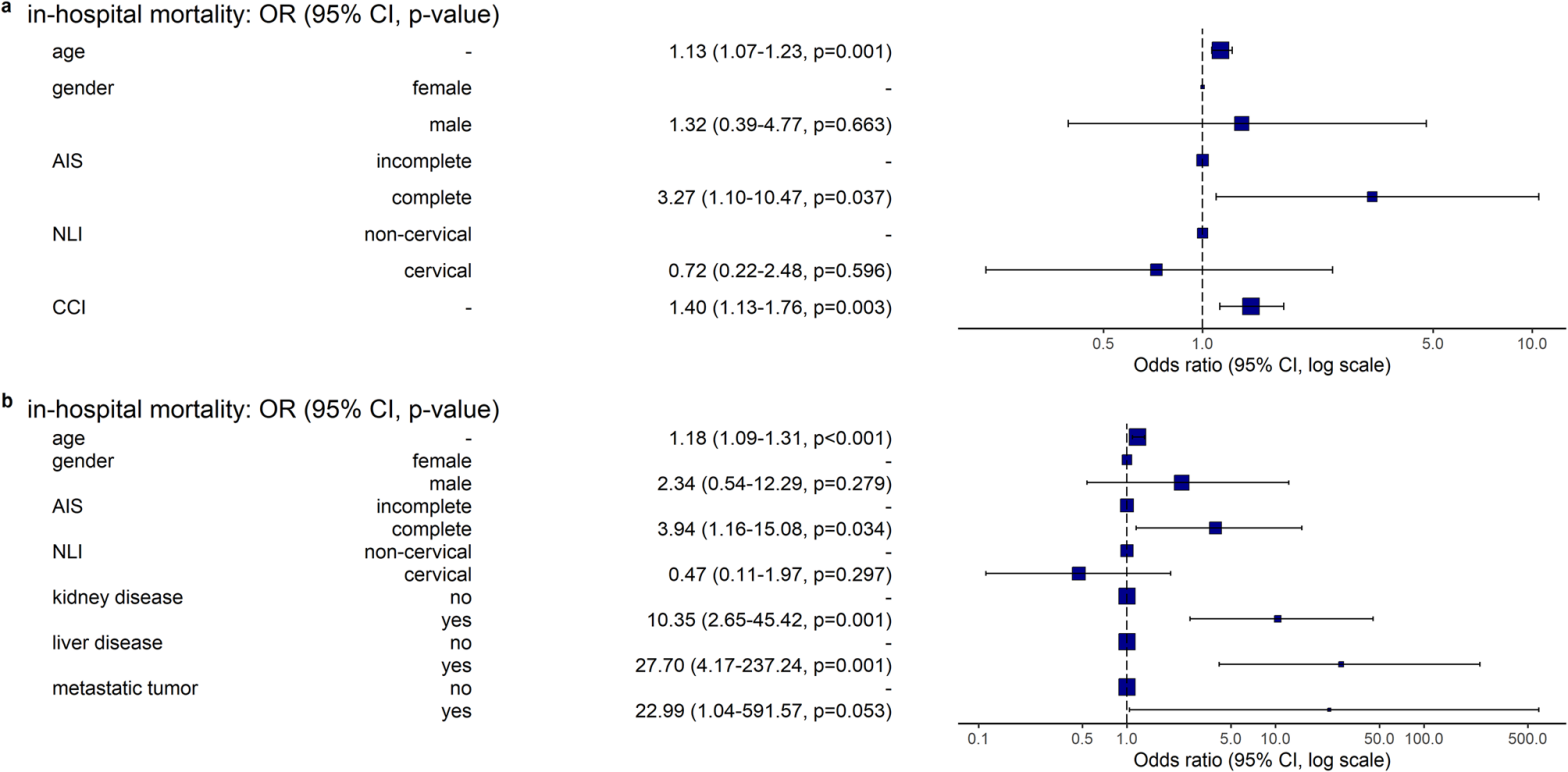
Multiple logistic regression results for in-hospital mortality using (**a**) baseline variables and Charlson score and (**b**) baseline- and identified predictor variables. The selection of predictor variables (**b**) is based on the conditional inference tree analysis. *AIS* ASIA Impairment Scale, *NLI* neurological level of injury, *CCI* Charlson Comorbidity Index, *OR* Odds ratio, *95% CI* 95% confidence interval (unadjusted for multiplicity).

The first regression analysis using age, gender, AIS, NLI and CCI as explanatory variables (Fig. 3a) suggests that the odds of in-hospital mortality increase by a factor of 3.27 (95% CI 1.10–10.47, p = 0.037) for patients with complete tSCI AIS A compared to incomplete tSCI AIS B–D, given all the other four predictors remain unchanged. Again, all else being equal, a one-year increase in age indicates an increase in odds by a factor of 1.13 (95% CI 1.07–1.23, p = 0.001), whereas an increase in the CCI by one point corresponds to an increase in odds by a factor of 1.40 (95% CI 1.13–1.76, p = 0.003).

In a second regression model (Fig. 3b) the CCI was replaced by the comorbidities kidney disease, liver disease and metastatic tumor as identified by the conditional inference tree algorithm. If all other six predictors remain unchanged, this model points to an increase in odds of in-hospital mortality by a factor of 1.18 (95% CI 1.09–1.31, p < 0.001) and 3.94 (95% CI 1.16–15.08, p = 0.034) with respect to age and AIS. As far as the comorbidities are concerned, the odds ratio estimates are 10.35 (95% CI 2.65–45.42, p = 0.001) for kidney disease, 27.70 (95% CI 4.17–237.24, p = 0.001) for liver disease, and 22.99 (95% CI 1.04–591.57, p = 0.053) for pre-existing metastatic tumor.

The odds ratios concerning the NLI and gender together with their confidence intervals did not reveal a clear effect on in-hospital mortality in our patient population in either of the two models.

### Clinical complications and causes of death after tSCI

The analysis of tSCI-specific clinical complications (Table 4) revealed an increased frequency of acute kidney injury (AKI) in the in-hospital deceased patients with 7 (35%) cases compared to 11 (3.65%) in the survivor group. Of these cases, irreversible AKI was solely observed in the deceased patient population with 4 out of 7 cases. Furthermore, increased frequencies of pneumonia, hyperthermia of unknown origin and bleeding during anticoagulant treatment were observed in in-hospital deceased patients. Cardiac events were also more frequent in these patients, whereas pressure ulcers and thromboembolic events were equally distributed between both patient groups. Conversely, urinary tract infections were more frequent in surviving patients.

**Table 4:** Clinical complications.

tSCI-specific complications	All patients N = 321	Survivors N = 301	Deceased N = 20	p-value
Cardiac complication	44 (13.7%)	39 (13.0%)	5 (25.0%)	0.169
Acute kidney injury (AKI)	18 (5.6%)	11 (3.7%)	7 (35.0%)	< 0.001
AKI reversible	14 (4.4%)	11 (3.7%)	3 (15.0%)	
AKI irreversible	4 (1.3%)	0 (0.0%)	4 (20.0%)	
Thromboembolic event	33 (10.3%)	31 (10.3%)	2 (10.0%)	1.000
Bleeding in combination with anticoagulation treatment	14 (4.4%)	11 (3.7%)	3 (15.0%)	0.048
Pneumonia	95 (29.6%)	83 (27.6%)	12 (60.0%)	0.005
Hyperthermia of unknown origin	41 (12.8%)	35 (11.6%)	6 (30.0%)	0.030
Urinary tract infection	240 (74.8%)	229 (76.1%)	11 (55.0%)	0.066
Pressure ulcer	99 (30.8%)	92 (30.6%)	7 (35.0%)	0.868

Absolute (N) and relative frequencies (%) of clinical complications. Data of acute kidney injury (AKI) are provided for the sum and fractions of reversible and irreversible cases.

The most important causes of death in the patient population were multiple organ failure (40%) and respiratory failure (35%) (Table 5). Thereby, multiple organ failure was often accompanied by septicemia and the clinical complications pneumonia and AKI. Furthermore, the occurrence of AKI was not restricted to patients suffering from pre-existing kidney disease. Additionally, cerebral hemorrhage (15%) and circulatory failure (10%) were identified causes of death (Table 5). Apparently, all cases of cerebral hemorrhage were associated with concomitant TBI and pre-existing kidney disease. This rare combination is observed in 2 out of 301 survivors (0.7%) but 3 out of 20 deceased patients (15%).

**Table 5:** Cause of death and associated characteristics of all in-hospital deceased patients.

Cause of death			Survival	Injury characteristics			Comorbid conditions				In-hospital complications			
Reason 1	Reason 2	EOL	Weeks after injury	AIS	NLI	TBI	CCI	Liver disease	Kidney disease	Metastatic tumor	Pneu-monia	Hyper-thermia	AKI	Bleeding & anticoagulation
Cerebral hemorrhage	Circulatory failure	No	1	A	Cervical	Yes	5	No	Yes	No	No	No	No	No
Cerebral hemorrhage	Circulatory failure	No	1	D	Cervical	Yes	2	No	Yes	No	No	No	No	Yes
Cerebral hemorrhage	Respiratory failure	Yes	15	C	Cervical	Yes	4	No	Yes	No	Yes	Yes	Yes (r)	Yes
Circulatory failure	Respiratory failure	Yes	2	A	Cervical	No	0	No	No	No	Yes	No	No	No
Circulatory failure	Pulmonary embolism	No	2	A	Lumbosacral	No	1	No	No	No	Yes	No	No	No
Multiple organ failure	Renal failure	Yes	14	C	Cervical	No	7	No	Yes	No	No	Yes	Yes (i)	No
Multiple organ failure	–	Yes	5	C	Lumbosacral	No	5	Yes	No	No	No	No	No	No
Multiple organ failure	Septicemia	No	19	A	Cervical	No	4	Yes	No	No	Yes	No	Yes (r)	No
Multiple organ failure	Septicemia	Yes	24	B*	Cervical*	No	8	No	No	Yes	Yes	Yes	No	No
Multiple organ failure	Septicemia	Yes	15	A	Thoracic	No	7	No	Yes	No	Yes	No	Yes (i)	No
Multiple organ failure	Septicemia	Yes	16	A	Thoracic	No	3	Yes	No	No	Yes	No	Yes (i)	No
Multiple organ failure	Septicemia	Yes	26	A	Thoracic	No	0	No	No	No	Yes	No	Yes (i)	No
Multiple organ failure	-	Yes	10	D	Cervical	No	1	No	No	No	No	Yes	No	No
Respiratory failure	Aspiration	No	12	A	Cervical	No	3	No	No	No	No	No	No	No
Respiratory failure	Septicemia	Yes	1	B	Lumbosacral	No	9	No	Yes	Yes	Yes	No	Yes (r)	No
Respiratory failure	–	Yes	12	A	Cervical	No	4	Yes	No	No	Yes	No	No	No
Respiratory failure	–	No	1	A	Cervical	No	3	No	Yes	No	No	No	No	No
Respiratory failure	–	Yes	12	D	Lumbosacral	No	1	No	No	No	No	No	No	Yes
Respiratory failure	–	Yes	5	A	Cervical	No	7	No	No	Yes	Yes	Yes	No	No
Respiratory failure	–	Yes	39	C	Cervical	No	5	No	Yes	No	Yes	Yes	No	No

If the cause of death was associated with more than one clinical condition, both were provided as reason 1 or 2. *AIS* ASIA impairment scale, *AKI* acute kidney injury, *(i)* irreversible, *(r)* reversible, *CCI* Charlson Comorbidity Index, *EOL* end of life decision, *NLI* neurological level of injury, *TBI* traumatic brain injury, *Assessment at admission impossible, AIS and NLI were determined during inpatient treatment.

Besides medical reasons, the death of 14 patients (70%) included end-of-life decisions. The median length of stay for deceased patients with a contributing end-of-life decision was 13 (IQR 6.25–15.75) weeks, compared to 1.5 (IQR 1.0–9.5) weeks for deceased patients without an end-of-life decision.

## Discussion

This retrospective cohort study adds tSCI specific data to the limited information available on acute in-hospital mortality. The observed in-hospital mortality amounts to 6.2% and is associated with a higher burden of premorbid conditions of the deceased patients. Pre-existing kidney or liver disease are identified as relevant predictors of in-hospital mortality and end-of-life decisions are recorded in 70% of the deceased cases.

The reported mortality rate of 6.2% parallels with information from comparable monocentric studies in Canada (5.7%^15^), Iceland (6.3%^20^) and the United States (6.6%^7^) and is also in the range of large nationwide epidemiological datasets (6.6%–7.5%^21^, 5.7%^22^), although the length of stay (LOS) of our cohort is expanded compared to most other studies, as it covers acute and early chronic rehabilitation phase. Patient characteristics reflect the changing epidemiology of tSCI in high income countries with an increase of fall related injuries of patients with high age and more frequent and multiple comorbid conditions. The univariate comparison clearly demonstrates that higher age and CCI are risk factors of in hospital mortality. Moreover, the frequency of falls and secondary referrals is higher in the deceased patient group. This may point to tSCI cases that are initially overlooked due to subtle neurological deficits^23^ or superimposed by pre-existing comorbidities. However, the time from injury to admission is only slightly increased and there is a high rate of AIS A injuries in the deceased patient group.

The univariate analysis of concomitant injuries revealed that none of the injuries were associated with in-hospital mortality. This complies with the high rate of a fall related etiology in deceased patients compared to the overall study population, so these patients are less likely to suffer concomitant injuries that are associated with higher energy trauma, e.g. traffic accidents.

The CCI, a sum score of 19 individual comorbidities weighted by their association with 1-year mortality, has shown validity in various clinical settings including chronic and acute tSCI^15,24^. Population-based analyses further confirmed that the number of comorbidities per patient correlates with in-hospital mortality in tSCI patients^16,17,25^. Our data also show a strong association of CCI-score with in-hospital mortality that also persists in a logistic regression model after adjustment for age, gender, AIS and NLI confirming related results^11^.

In addition, we extended the analysis from the overall CCI score to its contributing items and identified three pre-existing diseases as relevant predictors of in-hospital mortality. Especially pre-existing kidney disease and liver disease were identified in a conditional inference tree, exceeding all other comorbidities including metastatic tumor and age in their association with in-hospital mortality at the first splitting nodes of the tree. Odds ratios within the logistic regression model support their relevance for in-hospital mortality taking age, gender, AIS and NLI into account. Proteinuria and reduced creatinine clearance indicating an impaired kidney function have been identified as risk factor of mortality in 219 chronic SCI patients^26^ and preclinical and clinical results show an early decline in kidney function after tSCI^27,28^. In addition, a retrospective population-based study^29^ identified SCI patients that concurrently suffer from chronic kidney disease to have a shorter survival period and a higher 1-year mortality compared to patients without kidney disease. The association of pre-existing liver-disease with in-hospital mortality after tSCI in the present analysis has not been demonstrated before. Preclinical results from experimental SCI suggest that tSCI induces an acute liver pathology and a sustained liver inflammation^30–32^. This tSCI mediated liver pathology may potentiate pre-existing impairments of liver function, contributing to in-hospital mortality. Similarly, patients with traumatic brain injury and a concomitant liver cirrhosis have shown worse outcome and increased in-hospital mortality^33,34^.

In addition, the analysis of clinical complications demonstrates increased frequencies of AKI in the deceased patient group compared to survivors, with persistent AKI-cases solely present in the deceased patient group. Thereby, AKI was not restricted to patients with pre-existing kidney disease, indicating progression but also new onset of kidney dysfunction after tSCI. A study on AKI in critically ill patients^35^ identified septicemia and hypovolemia as most frequent AKI-etiologies, followed by the application of nephrotoxic drugs. The increased number of infections in the deceased patient group, including higher frequency of pneumonia and hyperthermia, and the development of septicemia present in some patients, most likely contribute to AKI formation. These results highlight the need of an early diagnosis and prevention of infections, which are a known risk factor for impaired recovery and mortality in the tSCI population^36,37^. In addition, a high degree (56%–87%) of patients with polypharmacy after tSCI is reported, especially in patients that are older and suffer from multiple illnesses^38–40^. Although beyond the scope of our analysis, we interpret the combination of pre-existing kidney disease and concomitant traumatic brain injury in patients died due to cerebral hemorrhage, as a subtle hint of altered drug metabolism and elimination. Light molecular weight heparin (LMWH), the major anticoagulant prophylaxis against the increased risk of thromboembolic events after tSCI, is known to accumulate in patients with kidney disease^41^, indicating the need of appropriate anticoagulation protocols and their close monitoring for the described patients.

Major causes of death in our patient population were multiple organ failure (MOF) followed by respiratory failure, cerebral hemorrhage and circulatory failure. A comparison with the existing literature is difficult due to the diverse leading causes of death provided^42^ and the limited availability of in-hospital mortality studies providing causes of death. However, like reported in other studies there is a high number of deaths due to respiratory failure^4,14,43–45^, but our results lack high numbers of death due to cardiac complications observed by these and other studies^15^. Moreover, we collected data on end-of-life decisions for all in-hospital deceased patients. Up to now, information on the contribution of end-of-life decisions after tSCI is scarce. Osterthun and colleagues^18^ found that end-of-life decisions are involved in 63% of cases, comparable to the result of 70% end-of-life cases within our study. The median length of stay indicates that end-of-life decisions were made after an appropriate time was left for an informed decision. End-of-life decisions represent a further reality belonging to the epidemiological change in the tSCI population and its reporting is recommended for upcoming studies on in-hospital mortality.

Although there is a high number of enrolled patients, the number of deceased patients of the study is, fortunately, rather low. Therefore, the quantified differences and odds ratios have to be interpreted cautiously and we emphasize the descriptive nature of this study. However, the finding of preexisting renal and liver comorbidities as associated with in-hospital mortality after tSCI is in line with multicenter evidence in the context of spine surgery^46^.

A further limitation of the study is based on the monocentric data collection. In consequence, death at the site of injury, during transport or primary treatment within other hospitals or after discharge are excluded from this analysis in contrast to large-scale population-based studies. On the other hand, the dataset provides almost complete clinical information of all tSCI-patients treated within a level I trauma center specialized in SCI care. High-resolution data includes information on premorbid conditions, clinical complications, clinical care and major injury characteristics. In combination with the increased length of stay at the study center, this dataset covers the range of acute to early chronic phases of tSCI. Thereby, the observational period covered, lies between most acute care studies that include the first days up to 1-month post-injury and larger epidemiological studies that cover the first year post-injury. Consistency with either kind of analyses from comparably industrialized countries has been shown, e.g. regarding the in-hospital mortality rate, the contribution of comorbidities, especially kidney disease, and end-of-life decisions to mortality after tSCI.

The present study emphasizes the importance of pre-existing comorbidities as predictor of in-hospital mortality after tSCI. It extends previous analyses on the contribution of comorbidities to in-hospital mortality by identifying relevant pre-existing illnesses. Based on CCI items, pre-existing kidney disease, liver disease and metastatic tumor were identified as predictors in a data driven analysis strategy. Awareness of specific premorbid health conditions allows an early identification of patients at risk for death during primary care, if access to medical history is available. The pre-existing impairment of kidney and liver, two organs most relevant for drug metabolism and elimination, provides opportunities to improve pharmaceutical treatment regimens, highly relevant for the growing population of geriatric tSCI-patients. In addition, the study confirms that apart from clinical parameters, a considerable proportion of in hospital deaths is associated with end-of-life decisions. Therefore, including its appropriate reporting will improve upcoming studies on in-hospital mortality after tSCI.

## Methods

### Study design and data source

The longitudinal observational study is based on the Comparative Outcome and Treatment Evaluation in Spinal Cord Injury (COaT-SCI) study^47^. Inpatient-study data are based on spinal cord injured patients aged 14 years and above, admitted to a specialized center for acute and rehabilitation care of traumatic and non-traumatic spinal cord injury at the level I trauma center, Trauma Hospital Berlin, Germany. Patients died at the site of injury, or during emergency transport were not included. In addition, death after discharge from initial hospitalization was not considered in this study. COaT-SCI-study data contains pre-injury medical status, injury characteristics, clinical course including complications and treatment regimen as well as socioeconomic and outcome parameters and spans from acute care to chronic stages of the disease. This analysis includes information of all patients admitted to the study center after suffering a tSCI from January 2011 to December 2017. Data was collected retrospectively using chart review of paper-based or electronic files. Source data verification was performed focusing on age and gender as well as on tSCI-specific items such as the American Spinal Injury Association Impairment Scale (AIS) and neurological level of injury (NLI), and tSCI associated major complications such as pulmonary infection, pressure ulcer, acute kidney injury (AKI) and mortality-associated parameters including cause of death and end-of-life decisions. The study was approved by the institutional ethics board of Charité-Universitätsmedizin Berlin (Approval-Number EA2/015/15) and adheres to the declaration of Helsinki and GCP-principles. Informed consent for the study participation was obtained from the participants or, in case of underage participants, from their legal representatives, prior to the inclusion in the Comparative Outcome and Treatment Evaluation in Spinal Cord Injury (COaT-SCI) study. In accordance with the regulations of the Berlin State Hospital Act, routinely collected clinical care data were used for non-commercial research in cases where it was not reasonable to obtain informed consent.

### Variable definitions

#### In-hospital death, cause of death and end-of-life decisions

The primary outcome variable “death during hospitalization” is defined as death occurring within the primary hospitalization at the Trauma Hospital Berlin after tSCI, including both, patients with primary and secondary referral to the clinical center.

The cause of death and accompanying diagnoses were recorded within the COaT-SCI study. Furthermore, decisions to withhold or withdraw potentially life-sustaining treatment (end-of-life decisions) of all in-hospital deceased patients were recorded. These include rejections of necessary medical treatment directly by the patient or her/his legal representative, a patient’s provision or ethical case discussions. Ethical case discussions were carried out in ethically controversial cases, they were led by an independent ethics expert and included all participating professions and family members, adjusting the therapeutic goals according to the supposed will of the patient.

#### Pre-injury illnesses, injury classification, concomitant injuries and clinical complications

Pre-injury illnesses were classified according to the Charlson Comorbidity Index (CCI) and the CCI is calculated as described earlier^48^. For baseline and outcome analysis of traumatic SCI, the International Standards for Neurological Classification of SCI (ISNCSCI) were applied^49^ and used to derive the NLI and AIS (AIS A–D). Documentation of concomitant injuries including fractures of the skull, the sternum/ribs, and the upper/lower extremities, traumatic brain injury, injury to the chest cavity, the abdominal cavity, to large blood vessels and to the *A. vertebralis* were based on clinical and radiological diagnostic criteria. Recording of tSCI-specific complications such as cardiac complications, thromboembolic events (deep vein thrombosis and/or pulmonary embolism), hyperthermia, pressure ulcer, and AKI were based on clinical, radiological and/or laboratory diagnostic criteria. AKI included patients requiring intermittent hemodialysis transiently (reversible AKI) or permanently (irreversible AKI). Urinary tract infections were defined based on the Centers for Disease Control and Prevention (CDC) criteria^50^. For the definition of pneumonia, the CDC criteria were modified in analogy to recent consensus definitions for a probable stroke associated pneumonia^51^.

#### Times and durations

The time and dates of injury, admission and discharge were recorded and used to calculate the respective durations, e.g. length of stay (LOS). Furthermore, the length of intensive-care-unit (ICU) stays was recorded.

### Statistical analysis

All statistical analyses were performed in the COaT-SCI database version as of December 4, 2019. Information on missing data is provided with the data-tables and complete cases were used for statistical analyses.

For our exploratory analysis, the study cohort was divided into two groups based on death during hospitalization. The distribution of continuous variables was described as median and quartiles, categorical variables were reported as absolute and relative frequencies. To compare continuous variables, the Kruskal–Wallis test was applied. Categorical data were compared using the Chi-square test. Whenever there were fewer than five observations per group, Fisher’s exact test was used. Exploratory p-values are reported as part of our descriptive analysis. Statistical analyses were carried out using R version 3.6.1^52^.

For our analysis on predictors of in-hospital mortality a conditional inference tree analysis was conducted based on the partykit package version 1.2–7^53^. We included the following predictor variables: age (continuous), gender (female/male), AIS (complete/incomplete), NLI (cervical/non-cervical), relevant items of the CCI (peripheral vascular disease, myocardial infarction, heart failure, cerebrovascular disease, dementia, chronic pulmonary disease, peptic ulcer, liver disease, diabetes mellitus, hemiplegia, kidney disease, metastatic tumor) and concomitant injuries (sternal-rib fracture, traumatic brain injury (TBI), vertebral arteria injury, chest injury, abdominal injury, upper extremity fracture). AIS and NLI were dichotomized as described because the cases of incomplete tSCI categories AIS B, C and D and thoracic and lumbosacral NLI were low in the deceased patient group. However, the binary variables still enable a differentiation according to injury severity and disability of the patients. Due to the low numbers of moderate or severe liver diseases (n = 1) and cases of diabetes with end-organ damage (n = 7) within the whole patient population, these cases were combined with cases of mild liver disease and diabetes without end-organ damage into the resulting variables liver disease or diabetes mellitus, respectively. All CCI-items and concomitant injury variables were included in the model as binary variables (no/yes). The model parameters mincriterion, minsplit and minbucket were set to 0.95, 20 and 4, respectively. For a comprehensive explanation of the algorithm, see reference^54^. Briefly, the regression tree is based on two consecutive steps: the association of each explanatory variable with in-hospital mortality is assessed on the p-value scale. The variable with the lowest p-value is selected and used to split the data set in such a way that the statistical discrepancy between the resulting child nodes is maximized. This procedure is repeated in each child node until the p-value exceeds the Bonferroni-adjusted significance level. Subsequently, in order to quantify the association of the selected variables with in-hospital mortality in a simultaneous manner, we performed a multiple logistic regression of in-hospital mortality including either the CCI-score or its items identified by the conditional inference tree. We report odds ratios along with confidence intervals and p-values, which have not been adjusted for multiplicity.

Since the stopping criterion of the algorithm is based on the Bonferroni adjusted significance level, we expect selection and post-selection bias to be negligible. However, as there are only 20 in-hospital deaths among the 321 patients, the results of the multiple logistic regressions need to be taken with considerable caution, as is also suggested by some very wide confidence intervals. Regression plots were created using the finalfit package for R, version 0.95^55^.

## Data Availability

The data analysed during the current study is not publicly available, due to legal restrictions under the General Data Protection Regulation of the European Union and other applicable national or local privacy regulations, but are available from the corresponding authors on reasonable request.

## Abbreviations

AIS: American Spinal Injury Association Impairment Scale
AKI: Acute kidney injury
BMI: Body mass index
CCI: Charlson Comorbidity Index
CDC: Centers for disease control and prevention
CI: Confidence interval
COaT-SCI: Comparative outcome and treatment evaluation in spinal cord injury
EOL: End-of-life
IQR: Interquartile range
LOS: Length of stay
NLI: Neurological level of injury
OR: Odds ratio
tSCI: Traumatic spinal cord injury

## References

[1] Bickenbach, J. *et al.* A global picture of spinal cord injury. in *International Perspectives on Spinal Cord Injury. WHO*. https://apps.who.int/iris/rest/bitstreams/441640/retrieve (2013).

[2] Shavelle RM, DeVivo MJ, Brooks JC, Strauss DJ, Paculdo DR (2015). Improvements in long-term survival after spinal cord injury?. Arch. Phys. Med. Rehabil..

[3] Devivo MJ (2012). Epidemiology of traumatic spinal cord injury: Trends and future implications. Spinal. Cord..

[4] Hagen EM, Lie SA, Rekand T, Gilhus NE, Gronning M (2010). Mortality after traumatic spinal cord injury: 50 years of follow-up. J. Neurol. Neurosurg. Psychiatry.

[5] Savic G (2017). Causes of death after traumatic spinal cord injury: A 70-year British study. Spinal Cord.

[6] O'Connor PJ (2005). Survival after spinal cord injury in Australia. Arch. Phys. Med. Rehabil..

[7] Casper DS (2018). Preinjury patient characteristics and postinjury neurological status are associated with mortality following spinal cord injury. Spine.

[8] Sabre L, Rekand T, Asser T, Korv J (2013). Mortality and causes of death after traumatic spinal cord injury in Estonia. J. Spinal Cord Med..

[9] Krassioukov AV, Furlan JC, Fehlings MG (2003). Medical co-morbidities, secondary complications, and mortality in elderly with acute spinal cord injury. J. Neurotrauma.

[10] Lau D (2019). Value of aggressive surgical and intensive care unit in elderly patients with traumatic spinal cord injury. Neurosurg. Focus.

[11] Inglis T (2020). In-hospital mortality for the elderly with acute traumatic spinal cord injury. J. Neurotrauma.

[12] Furlan JC, Fehlings MG (2009). The impact of age on mortality, impairment, and disability among adults with acute traumatic spinal cord injury. J. Neurotrauma.

[13] Neumann CR, Brasil AV, Albers F (2009). Risk factors for mortality in traumatic cervical spinal cord injury: Brazilian data. J. Trauma.

[14] Lidal IB (2007). Mortality after spinal cord injury in Norway. J. Rehabil. Med..

[15] Furlan JC, Kattail D, Fehlings MG (2009). The impact of co-morbidities on age-related differences in mortality after acute traumatic spinal cord injury. J. Neurotrauma.

[16] Selassie AW, Varma A, Saunders LL, Welldaregay W (2013). Determinants of in-hospital death after acute spinal cord injury: A population-based study. Spinal Cord.

[17] Varma A, Hill EG, Nicholas J, Selassie A (2010). Predictors of early mortality after traumatic spinal cord injury: A population-based study. Spine.

[18] Osterthun R, van Asbeck FW, Nijendijk JH, Post MW (2016). In-hospital end-of-life decisions after new traumatic spinal cord injury in the Netherlands. Spinal Cord.

[19] DeVivo MJ, Biering-Sorensen F, New P, Chen Y (2011). Standardization of data analysis and reporting of results from the International Spinal Cord Injury Core Data Set. Spinal Cord.

[20] Knutsdottir S (2012). Epidemiology of traumatic spinal cord injuries in Iceland from 1975 to 2009. Spinal Cord.

[21] Jain NB (2015). Traumatic spinal cord injury in the United States, 1993–2012. JAMA.

[22] Shibahashi K, Nishida M, Okura Y, Hamabe Y (2019). Epidemiological state, predictors of early mortality, and predictive models for traumatic spinal cord injury: A multicenter nationwide cohort study. Spine.

[23] Lam C (2017). Risk factors for 14-day rehospitalization following trauma with new traumatic spinal cord injury diagnosis: A 10-year nationwide study in Taiwan. PLoS ONE.

[24] Rochon PA (1996). Comorbid illness is associated with survival and length of hospital stay in patients with chronic disability: A prospective comparison of three comorbidity indices. Med. Care.

[25] Boakye M (2008). Laminectomy and fusion after spinal cord injury: national inpatient complications and outcomes. J. Neurotrauma.

[26] Greenwell MW, Mangold TM, Tolley EA, Wall BM (2007). Kidney disease as a predictor of mortality in chronic spinal cord injury. Am. J. Kidney Dis..

[27] Rodriguez-Romero V, Guizar-Sahagun G, Castaneda-Hernandez G, Reyes JL, Cruz-Antonio L (2018). Early systemic alterations in severe spinal cord injury: An experimental study on the impact of injury level on renal function. Spine.

[28] Pettersson-Hammerstad K, Jonsson O, Svennung IB, Karlsson AK (2008). Impaired renal function in newly spinal cord injured patients improves in the chronic state–effect of clean intermittent catheterization?. J. Urol..

[29] Yu SC (2017). One-year mortality of patients with chronic kidney disease after spinal cord injury: A 14-year population-based study. World Neurosurg..

[30] Goodus MT, Sauerbeck AD, Popovich PG, Bruno RS, McTigue DM (2018). Dietary green tea extract prior to spinal cord injury prevents hepatic iron overload but does not improve chronic hepatic and spinal cord pathology in rats. J. Neurotrauma.

[31] Sauerbeck AD (2015). Spinal cord injury causes chronic liver pathology in rats. J. Neurotrauma.

[32] Hundt H (2011). Assessment of hepatic inflammation after spinal cord injury using intravital microscopy. Injury.

[33] Lustenberger T (2011). Liver cirrhosis and traumatic brain injury: A fatal combination based on National Trauma Databank analysis. Am. Surg..

[34] Talving P (2013). The impact of liver cirrhosis on outcomes in trauma patients: A prospective study. J. Trauma Acute Care Surg..

[35] Hoste EA (2015). Epidemiology of acute kidney injury in critically ill patients: The multinational AKI-EPI study. Intensive Care Med..

[36] Failli V (2012). Functional neurological recovery after spinal cord injury is impaired in patients with infections. Brain.

[37] Kopp MA (2017). Long-term functional outcome in patients with acquired infections after acute spinal cord injury. Neurology.

[38] Guilcher SJT (2018). Prescription drug claims following a traumatic spinal cord injury for older adults: A retrospective population-based study in Ontario, Canada. Spinal Cord.

[39] Hwang M, Zebracki K, Vogel LC (2015). Medication profile and polypharmacy in adults with pediatric-onset spinal cord injury. Spinal Cord.

[40] Hope ME, Kailis SG (1998). Medication usage in a spinal cord injured population. Spinal Cord.

[41] Lim W (2008). Low-molecular-weight heparin in patients with chronic renal insufficiency. Intern. Emerg. Med..

[42] Chamberlain JD, Meier S, Mader L, von Groote PM, Brinkhof MW (2015). Mortality and longevity after a spinal cord injury: Systematic review and meta-analysis. Neuroepidemiology.

[43] Daneshvar P (2013). Spinal cord injuries related to cervical spine fractures in elderly patients: Factors affecting mortality. Spine J..

[44] Leal-Filho MB (2008). Spinal cord injury: epidemiological study of 386 cases with emphasis on those patients admitted more than four hours after the trauma. Arq. Neuropsiquiatr..

[45] Sokolowski MJ, Jackson AP, Haak MH, Meyer PR, Sokolowski MS (2007). Acute mortality and complications of cervical spine injuries in the elderly at a single tertiary care center. J. Spinal Disord. Tech..

[46] Kushioka J (2020). Risk factors for in-hospital mortality after spine surgery: A matched case-control study using a multicenter database. Spine J..

[47] The COaT-SCI Register. https://www.coat-sci.org/home-en.html. (2020).

[48] Charlson ME, Pompei P, Ales KL, MacKenzie CR (1987). A new method of classifying prognostic comorbidity in longitudinal studies: Development and validation. J. Chronic Dis..

[49] Kirshblum SC (2011). Reference for the 2011 revision of the international standards for neurological classification of spinal cord injury. J. Spinal Cord Med..

[50] Horan TC, Andrus M, Dudeck MA (2008). CDC/NHSN surveillance definition of health care-associated infection and criteria for specific types of infections in the acute care setting. Am. J. Infect. Control.

[51] Smith CJ (2015). Diagnosis of stroke-associated pneumonia: recommendations from the pneumonia in stroke consensus group. Stroke.

[52] R Core Team. *R: A Language and Environment for Statistical Computing*. https://www.R-project.org/. (2019).

[53] Hothorn T, Zeileis A (2015). partykit: A modular toolkit for recursive partytioning in R. J. Mach. Learn. Res..

[54] Tanadini LG (2015). Toward inclusive trial protocols in heterogeneous neurological disorders: Prediction-based stratification of participants with incomplete cervical spinal cord injury. Neurorehabil. Neural. Repair..

[55] Harrison, E., Drake, T. & Ots, R. *finalfit: Quickly Create Elegant Regression Results Tables and Plots when Modelling*. R package version 0.9.5. https://CRAN.R-project.org/package=finalfit. (2019).

